# Complex repolarization dynamics in *ex vivo* human ventricles are independent of the restitution properties

**DOI:** 10.1093/europace/euad350

**Published:** 2023-11-25

**Authors:** Shahriar Iravanian, Ilija Uzelac, Anand D Shah, Mikael J Toye, Michael S Lloyd, Michael A Burke, Mani A Daneshmand, Tamer S Attia, John David Vega, Mikhael F El-Chami, Faisal M Merchant, Elizabeth M Cherry, Neal K Bhatia, Flavio H Fenton

**Affiliations:** Department of Medicine, Division of Cardiology, Emory University School of Medicine, 1364 Clifton Road, Atlanta, GA 30322, USA; Georgia Institute of Technology, Department of Physics, 837 State St NW, Atlanta, GA 30332, USA; Department of Medicine, Division of Cardiology, Emory University School of Medicine, 1364 Clifton Road, Atlanta, GA 30322, USA; Georgia Institute of Technology, Department of Physics, 837 State St NW, Atlanta, GA 30332, USA; Department of Medicine, Division of Cardiology, Emory University School of Medicine, 1364 Clifton Road, Atlanta, GA 30322, USA; Department of Medicine, Division of Cardiology, Emory University School of Medicine, 1364 Clifton Road, Atlanta, GA 30322, USA; Department of Surgery, Division of Cardiovascular Surgery, Emory University School of Medicine, 1364 Clifton Road, Atlanta, GA 30322, USA; Department of Surgery, Division of Cardiovascular Surgery, Emory University School of Medicine, 1364 Clifton Road, Atlanta, GA 30322, USA; Department of Surgery, Division of Cardiovascular Surgery, Emory University School of Medicine, 1364 Clifton Road, Atlanta, GA 30322, USA; Department of Medicine, Division of Cardiology, Emory University School of Medicine, 1364 Clifton Road, Atlanta, GA 30322, USA; Department of Medicine, Division of Cardiology, Emory University School of Medicine, 1364 Clifton Road, Atlanta, GA 30322, USA; Georgia Institute of Technology, Department of Physics, 837 State St NW, Atlanta, GA 30332, USA; Department of Medicine, Division of Cardiology, Emory University School of Medicine, 1364 Clifton Road, Atlanta, GA 30322, USA; Georgia Institute of Technology, Department of Physics, 837 State St NW, Atlanta, GA 30332, USA

**Keywords:** Ventricular fibrillation, Optical mapping, Heart transplantation, Nonlinear dynamics, Signal processing

## Abstract

**Aims:**

The mechanisms of transition from regular rhythms to ventricular fibrillation (VF) are poorly understood. The concordant to discordant repolarization alternans pathway is extensively studied; however, despite its theoretical centrality, cannot guide ablation. We hypothesize that complex repolarization dynamics, i.e. oscillations in the repolarization phase of action potentials with periods over two of classic alternans, is a marker of electrically unstable substrate, and ablation of these areas has a stabilizing effect and may reduce the risk of VF. To prove the existence of higher-order periodicities in human hearts.

**Methods and results:**

We performed optical mapping of explanted human hearts obtained from recipients of heart transplantation at the time of surgery. Signals recorded from the right ventricle endocardial surface were processed to detect global and local repolarization dynamics during rapid pacing. A statistically significant global 1:4 peak was seen in three of six hearts. Local (pixel-wise) analysis revealed the spatially heterogeneous distribution of Periods 4, 6, and 8, with the regional presence of periods greater than two in all the hearts. There was no significant correlation between the underlying restitution properties and the period of each pixel.

**Conclusion:**

We present evidence of complex higher-order periodicities and the co-existence of such regions with stable non-chaotic areas in *ex vivo* human hearts. We infer that the oscillation of the calcium cycling machinery is the primary mechanism of higher-order dynamics. These higher-order regions may act as niduses of instability and may provide targets for substrate-based ablation of VF.

## Graphical Abstract

**Graphical Abstract:**
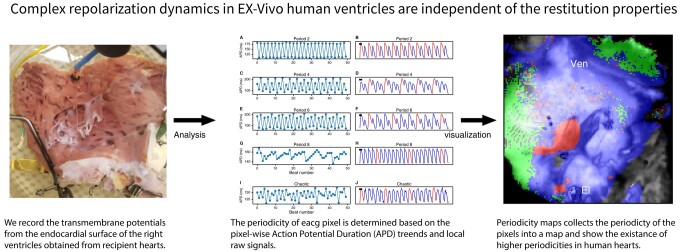

**What’s new?**

- We report the first detection of higher-order periodicity in the repolarization phase of action potentials recorded from human ventricles.
- We detect stable Period-4 and quasi-stable Periods 6 and 8 in human hearts.
- The underlying electrophysiological properties, measured by the restitution curve and local conduction velocity, do not predict the repolarization dynamics when pacing at faster rates.
- Higher-order regions may point to electrically unstable substrates and can potentially be a target of ablation.

## Introduction

Malignant ventricular arrhythmias, including polymorphic ventricular tachycardia (VT) and ventricular fibrillation (VF), are the leading causes of sudden cardiac death. However, the mechanisms of transition from a regular rhythm to chaotic VF are still not well understood.^1–3^

The concordant to discordant action potential duration (APD) alternans pathway to VF initiation is one of the most extensively studied mechanisms of VF initiation.^4–6^ The APD alternans is the beat-to-beat (Period-2) oscillation in the APD and is the simplest quantifier of repolarization dynamics.^7^ According to the concordant to discordant alternans pathway, the heart is in a meta-stable but not chaotic condition just before VF initiation, waiting for a timely trigger to exploit the large repolarization gradient caused by discordant alternans to degenerate regular rhythms into chaotic fibrillation.^8,9^

Understanding the mechanisms of ventricular tachyarrhythmias and their initiation is not just of theoretical interest but has practical implications. Ablation of monomorphic VT is the cornerstone therapy for drug-refractory VT or VT storm; conversely, ablation of VF substrates is in its infancy. Ventricular fibrillation ablation is performed rarely and only in specific circumstances targeting triggers or fixed anatomical structures, such as the right ventricular outflow tract ablation in Brugada syndrome.^10–12^ However, due to the lack of detailed mechanistic insight into the initiation of VF, functional substrate ablation of VF is still not possible.

Despite its central mechanistic position, alternans *per se* cannot guide ablation since most cardiac tissues exhibit the APD alternans when stimulated at a sufficiently fast rate. The hope is that by moving beyond alternans to more complex dynamics, which are specific to diseased myocardium, we may gain useful localizing information to find unstable substrates suitable for ablation. Therefore, one of the motivations of the current study is the hypothesis that *the electrical instability starts focally before expanding to the entire ventricles, and ablation of these areas has a stabilizing effect and may reduce the risk of VF*.

Classic alternans with Period-2 results from the first bifurcation in a cascade of bifurcations with periods of 2, 4, 8, and higher powers-of-two. We anticipate that as the cycle length becomes shorter, higher-order periods appear until the system transitions to chaos and, hence, VF. The intermediate stages in the period-doubling cascade in cardiac tissue have been experimentally elusive and are reported in only a few animal models but not in human hearts.^13–15^

Before directly testing the feasibility of VF ablation, we need to verify the premise of the hypothesis, namely the existence and detectability of electrically unstable substrates before VF induction. We use complex repolarization dynamics, i.e. higher-order periodicities, as a surrogate for electrical instability. Specifically, we try to answer the following questions:

1. Do higher-order periods (> Period-2) occur in (diseased) human hearts?
2. What is the spatial distribution of the higher-order areas (are they focal)?
3. Are the baseline electrophysiological characteristics predictive of the local dynamics?

## Methods

### Heart harvesting and preparation

The study protocol was approved by the Emory University and Georgia Institute of Technology Institutional Review Boards (IRB). Patients consented to the research protocol before surgery. We obtained hearts from the recipients of orthotopic heart transplantation at Emory University Hospital. At the time of surgery, each patient was fully heparinized and placed on a cardiopulmonary bypass machine after circulatory arrest was induced with the infusion of cold cardioplegia solution. Then, the pericardium was opened, and the recipient’s heart was removed using the bicaval technique.

Within 5 min of heart harvesting, the explanted heart was perfused from the arteries with cold cardioplegia solution for 5 min and transported to the optical-mapping lab. Once in the lab, the left main and right coronary arteries were cannulated, and the heart was perfused with warm (37°C) oxygenated Tyrode’s solution until the return of the spontaneous contractions. The heart was placed in an imaging chamber and perfused for at least half an hour to recover from the cardioplegia. Before optical mapping, the cardiac motion was suppressed with the help of the reversible myosin II ATPase inhibitor (−)-Blebbistatin.^16^ The hearts were immobilized with a bolus of blebbistatin at a concentration of 10 μM. The concentration was reduced to a maintenance level of 1.8 μM for the duration of optical mapping.^17^ However, if contraction was not completely suppressed, the maintenance concentration was increased to a maximum of 3.6 μM. In some cases, a transparent mechanical restrainer was placed on the tissue to reduce contraction artefacts further. Compared with the first-generation excitation-contraction uncouplers like 2,3-butanedione monoxime, blebbistatin has a minimum effect on calcium cycling, especially at low concentrations. Blebbistatin may prolong the APD,^17^ but it is unclear whether this is a primary effect or the result of lower ischaemia due to less oxygen demand.^18^

### Optical mapping

Optical mapping is one of the main tools to study complex arrhythmias.^19^ Staining arterially perfused explanted whole heart or a segment with voltage-sensitive fluorescent dyes allows for non-contact mapping of the transmembrane potential with high spatial (sub-millimeter) and temporal (milliseconds) resolutions.

In this study, after the heart was prepared as earlier (arterially cannulated, perfused, and immobilized), it was stained with 1 mg of near-infrared voltage-sensitive dye JPW-6003 (also known as di-4-ANBDQPQ) dissolved in ethanol.^20,21^ The first voltage measurements were obtained from the epicardial surface as long as there was an acceptable imaging window (depending on the amount of fat across the epicardial surface). Afterward, the right ventricular free wall was cut along a line adjacent to the interventricular septum, and the major arterial bleeders, in particular the distal right coronary artery and the conus branch, were ligated to maintain a perfusion pressure of 60–70 mmHg. We report on the results of transmembrane potential mapping from the endocardial surface of the right ventricles.

The tissue was excited using a deep red LED coupled with a 660/20 nm bandpass filter, and the emitted fluorescence from a square area with a side of ∼7 cm (range, 6–8 cm) was directed through a long pass 700 nm filter into an Electron-Multiplying-Charged-Coupled Camera (EMCCD). Image acquisition was performed at 500 Hz and with a resolution of 128 × 128 pixels.

### Study protocol

The study protocol was organized as multiple restitution runs. In each run, the right ventricles were paced at progressively faster rates, starting at a pacing cycle length of ∼2000 ms and down to either the emergence of 2:1 block or induction of a re-entrant arrhythmia (VT or VF). Optical signals were recorded from the endocardial surface in 20–40 s segments. We compared the signal recorded just before VT/VF induction (while still global 1:1 capture and conduction were present) to a control recording at 500 ms to detect higher-order periods.

### Signal processing

The transmembrane potential data for each pixel is low-passed filtered by a cut-off of 50 Hz, and the output is normalized in the 0–1 range. Spatial smoothing is performed by applying a variational method (see Supplementary material online, *SA*).

Period-4 and higher-order dynamics are not distributed uniformly over the recording area.^13^ Instead, they are localized to a few regions. In response, we have developed two complementary processing pathways, one *global* to detect low-amplitude higher-order periodic signals from the whole imaging area and the other *local* to localize Period-4 and higher at a pixel level.

**Figure 1:**
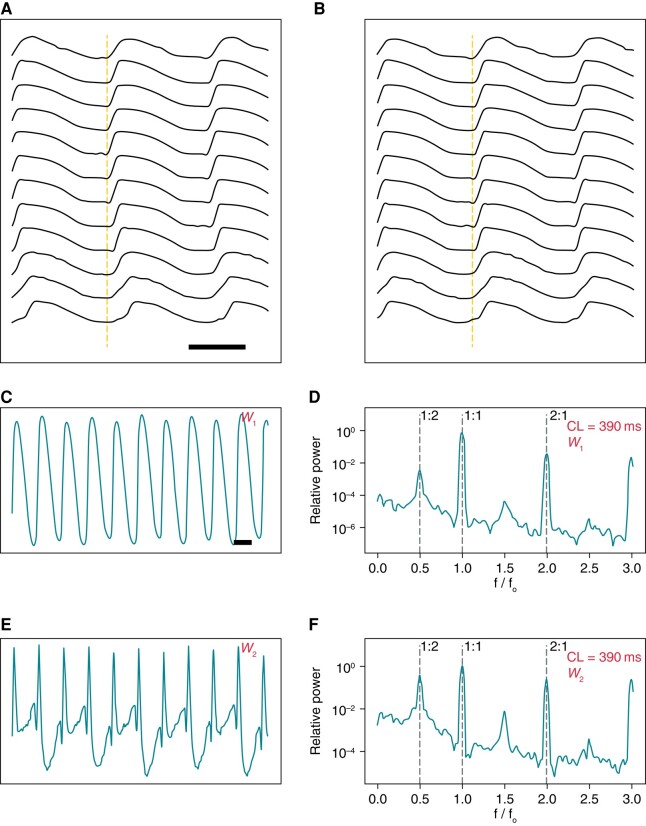
(*A*) The schematics of global analysis. Spatiotemporally processed signals recorded from multiple points on a line, showing staggered action potentials upstrokes consistent with wavefront propagation. (*B*) Same signals as *A* shifted to align the upstrokes. (*C*) The first principle component ( $W_{1}$) displays alternans. (*D*) Spectrogram of *C*, showing a 1:2 peak of alternans. The second principle component ( $W_{2}$) displays more pronounced alternans larger than *C* (*E*). Spectrogram of *E*, shows a prominent 1:2 peak of alternans (*F*). The bar in *A* depicts 200 ms.

*Figure 1* explains the global analysis methodology (details in Supplementary material online, *SB*). The output of the global analysis is a spectrogram, where the frequency is normalized to the pacing frequency, such that the 1:1 peak corresponds to the principal action potential propagation. We are mainly interested in the sub-harmonics of the 1:1 peak. The 1:2 peak (located at exactly half the driving frequency) is a sign of Period-2 alternans. Similarly, the 1:4 peak is a marker of the Period-4 oscillation in the repolarization phase.

In addition to global analysis, we apply local analysis to find the dominant periodicity (i.e. the most common period) of each pixel expressed as an integer in the range 1–8. The local analysis uses a combinatorial algorithm, which is described in Supplementary material online, *SC*.^22^

### Restitution curve

**Figure 2:**
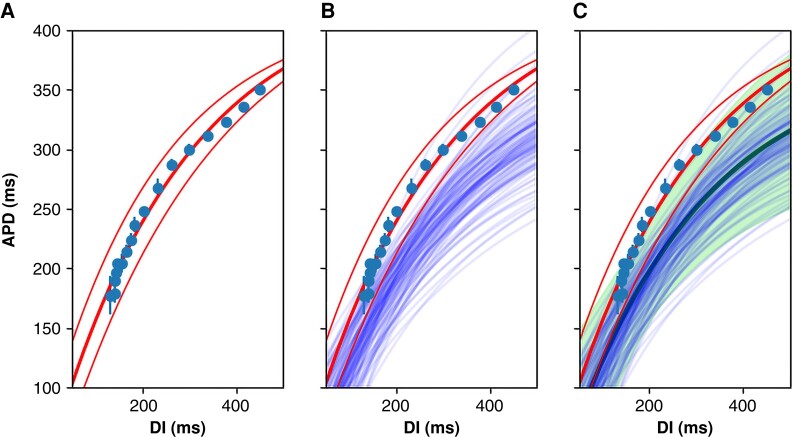
The schematic of composite restitution-curve generation. An exponential curve fits a set of (DI, APD) data points for one pixel. (*A*) The 95% confidence-interval curves are also shown. (*B*) The restitution curves for all the pixels in a region of interest (say, with Period-2 on the local analysis) are calculated (only 100 curves are shown here). (*C*) Monte Carlo sampling is performed to generate a composite restitution curve (the thick central line) and the confidence interval (shown as a continuum). APD, action potential duration; DI, diastolic interval.

According to the restitution hypothesis, the APD is a function of the preceding diastolic interval (DI). The electrophysiological characteristics of each pixel are evaluated by calculating the local restitution curve by fitting an exponential curve to the (DI, APD) data points obtained from pacing the heart at different cycle lengths (*Figure 2A*). The exponential curve is parametrized as,


$$\mathrm{APD}(\mathrm{DI})=\mathrm{AP}D^{\infty }(1-e^{(\mathrm{DI}-DI_{0})/\tau })$$

where $\mathrm{AP}D^{\infty }$ is the APD achieved at long cycle lengths, $DI_{0}$ is the interception of the exponential curve and the *x*-axis, and *τ* is the time-constant measuring the steepness of the restitution curve. There are multiple definitions for restitution curves in the literature.^23^ Here, we use dynamic or steady-state restitution based on the measurements of APD and DI during stimulation at a constant cycle length for hundreds of beats to reach a steady-state condition. *Figure 2B* and *C* shows how to combine multiple restitution curves to derive a composite curve with confidence intervals.

### Conduction velocity

Pixel-wise conduction velocity is calculated from the smoothed activation map, i.e. the map showing the time of the upstroke of action potentials for each pixel. Specifically, the conduction velocity is inversely proportional to the magnitude of the gradient of the activation time.

## Results

### The general characteristics of the hearts

We report on six explanted human hearts (designated H1–H6) removed during heart transplantation surgery. Demographics and clinical history of the hearts are presented in *Table 1*. The aggregate electrophysiological properties of each heart, as described by the restitution-curve parameters, are presented in *Table 2*.

**Table 1:** The baseline characteristics of the hearts

Heart	Age/sex	Diagnosis	Ejection fraction (%)	Amiodarone	Inotropes	LVAD
H1	54/M	Hypertrophic cardiomyopathy	30	No	Yes	No
H2	38/F	Non-compaction	10	No	Yes	No
H3	60/F	Doxorubicin toxicity	10	Yes	Yes	No
H4	56/M	Idiopathic	20	Yes	Yes	No
H5^a^						
H6	40/M	Idiopathic	10	Yes	No	Yes

LVAD, left ventricular assist device.

^a^Rejected donor heart.

**Table 2:** The electrophysiological characteristics (restitution-curve parameters) of the hearts

Heart	$\mathrm{AP}D^{\infty }$	$DI_{0}$	*τ*
H1	383 ± 43	−18 ± 52	302 ± 79
H2	300 ± 26	−84 ± 42	318 ± 65
H3	289 ± 30	111 ± 59	69 ± 47
H4	351 ± 29	−72 ± 40	421 ± 68
H5	261 ± 71	124 ± 90	120 ± 100
H6	419 ± 24	−51 ± 17	374 44

### Global analysis detects 1:4 peaks

Three hearts (H1–H3) showed a prominent and statistically significant ( >3σ) 1:4 peak. A borderline peak of uncertain significance was seen in another one (H4). No 1:4 peak was present in two hearts (H5 and H6).

**Figure 3:**
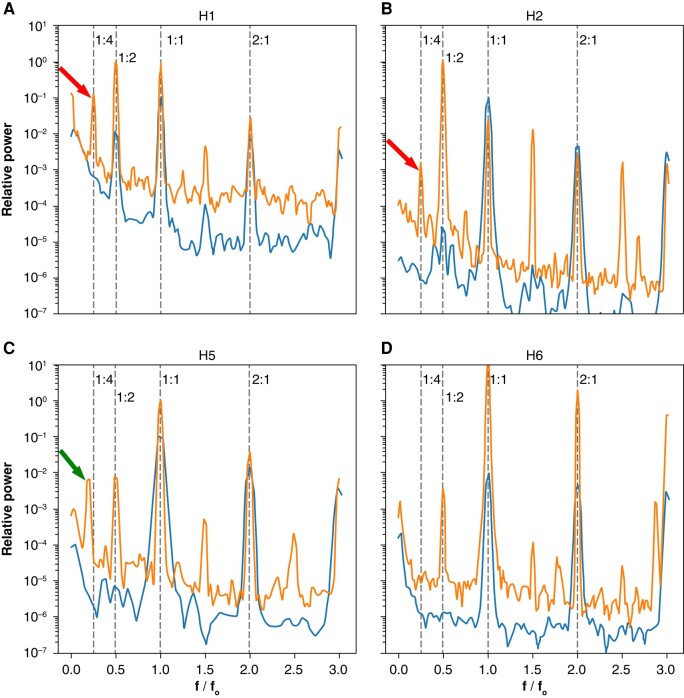
Comparison of baseline and pre-VF spectrograms using global analysis. The lower spectrograms are the baseline (stimulation cycle length of 500 ms except for H4 at 800 ms) and the upper spectrograms are obtained just before VF induction. (*A* and *B*) H1, H2 exhibit prominent 1:4 peaks, while no discernable 1:4 peak is seen for (*D*) H6. H4 has a ∼0.18 peak, corresponding to mainly (*C*) Period-6 activity. The baseline signals are multiplied by 0.1 to offset the signals for better visualization.

*Figure 3A* depicts the global spectrogram of H1. The baseline spectrogram at 500 ms shows the expected 1:1 peak (the primary activation) and a small 1:2 peak, signifying repolarization alternans. As the heart was stimulated faster at a cycle length of 310 ms, the 1:2 peak became larger, and a new 1:4 peak emerged. This means that a bifurcation occurred somewhere between 500 and 310 ms, and the global dynamics had Period-4 periodicity. Pacing this heart faster at 300 ms resulted in VF.

H2 follows a similar pattern (panel *B*). Again, we observed barely discernible alternans at 500 ms with a strong 1:2 peak and a clear 1:4 peak at 270 ms. Similarly, VF was induced while pacing at 260 ms (*Figure 3*).

The peak of interest in H5 (panel *C*) is offset from the 1:4 location and is around ∼0.18. This peak is further discussed below.

H6 is a negative example with no 1:4 peak (panel *D*). There is a prominent 1:2 alternans peak while stimulating at 270 ms, but no significant peak at 1:4. Stimulating this heart faster resulted in a conduction block but no re-entrant arrhythmia.

**Figure 4:**
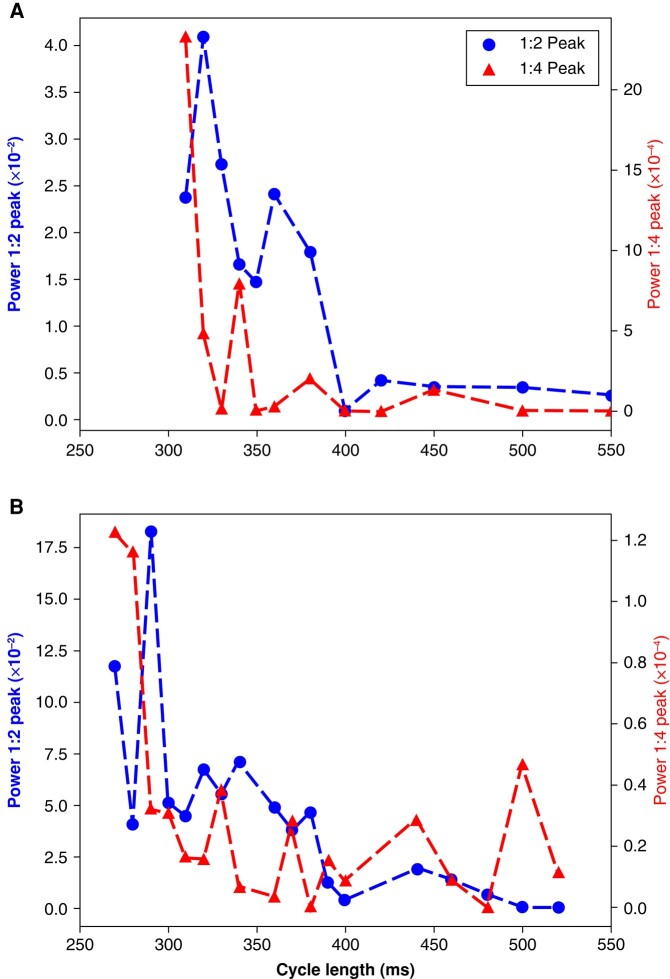
The pacing frequency dependence of 1:2 and 1:4 peaks. In both panels, the 1:2 peak (the classic APD alternans) starts when the cycle length decreases to ∼400 ms. On the other hand, the 1:4 peak only rises above the baseline once the cycle length decreases to 300–350 ms. Also, note the different scaling of the 1:4 peak compared with the 1:2 peak, which is 2–3 orders of magnitude smaller than the 1:2 peak. These results significantly reduce the chance that the observed 1:4 peaks are processing artefacts and point to their dynamical origin.

The absence of the 1:4 peak at 500 ms in hearts with a prominent 1:4 peak at short cycle lengths significantly reduces the chance that this peak is a processing artefact and points to its dynamic origin. We can probe the dynamics further by looking at the stimulation frequency dependencies of the 1:2 and 1:4 peaks (*Figure 4*).

### Visualizing higher-periodicity signals

**Figure 5:**
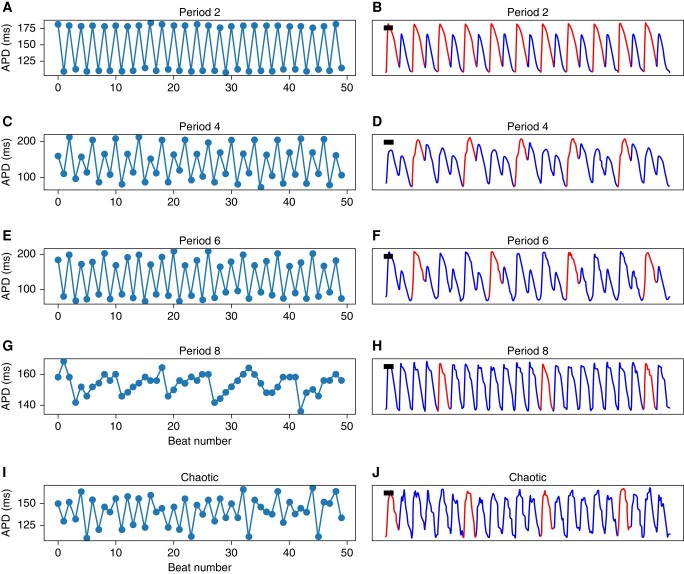
Examples of signals with different periodicities. Representative pixel-level optical-mapping signals with (*A* and *B*) Period-2, (*C* and *D*) Period-4, (*E* and *F*) Period-6, (*G* and *H*) Period-8, and (*I* and *J*) higher-order/chaotic are shown. Note the intermittency of Period-8. APD, action potential duration.

*Figure 5* lists representative examples of Period-2, Period-4, Period-6, Period-8, and higher-order chaotic signals and the corresponding APD trends. Period-4 is stable, but higher-order periods are intermittent. Specifically, the Period-8 signal (*Figure 5G* and *H*) is irregular but still has sufficient periodicity to be annotated Period-8 by the local algorithm.

### Local analysis detects both Period-4 and higher

**Figure 6:**
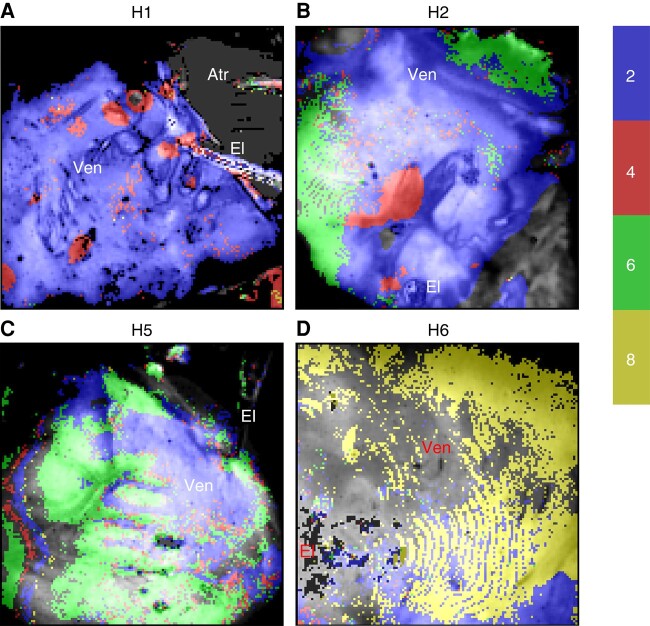
The distribution of higher-order areas. (*A*) In H1, there are multiple Period-4 areas in a sea of classic alternans. (*B*) H2 shows both 1:4 and 1:6 areas. (*C*) H5 has large areas of Period-6 without significant Period-4. (*D*) The main feature of H6 is a large area of Period-8 without significant 1:2 or 1:4 regions . The background images represent anatomy. The pixels are colored based on their periodicitiy according to the colorbar on the right. Atr, atrial; El, pacing electrode; Ven, ventricles.

The distribution of areas with higher-order periodicity is heterogeneous in both space and time and is variable in different hearts. The spatial distribution of the dominant periodicity in four hearts is depicted in *Figure 6*.

H1, which shows a dominant 1:4 peak in global analysis, has areas of stable Period-4 periodicity localized to a few discrete islands with roughly circular borders (*Figure 6A*). The rest of the ventricle exhibits Period-2 with no significant Period-6 or higher. On the other hand, H5, which does not have a prominent 1:4 peak, has large areas of Period-6 but no significant amount of Period-2 (*Figure 6C*). H2 is a mixed case with both islands of Period-2 and large areas of Period-6 (*Figure 6B*). H6 is unusual in having large regions of Period-8 (*Figure 5D*); however, it should be noted that H6 is the only heart in our study that was on a left ventricular assist device before transplantation (*Table 1*).

**Figure 7:**
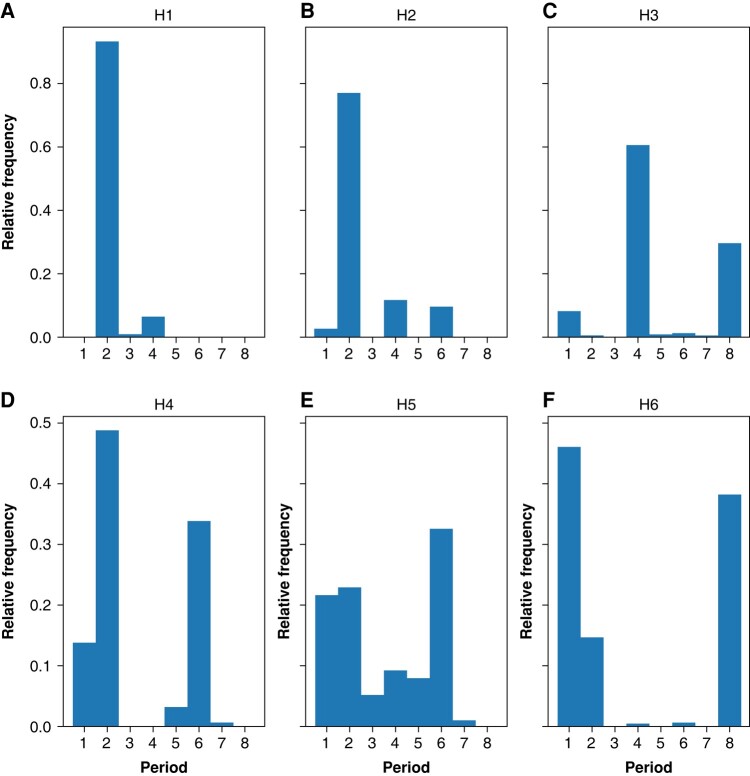
The relative frequency of different periodicities. Each histogram shows the relative proportions of pixels with a given period (in the range of 1–8) for each of the six hearts.

*Figure 7* displays the relative proportion of pixels of different periodicities in the six study hearts. Again, we noted the significant variability among the hearts. The results are consistent with the global analysis. For example, H1–H3 have clear 1:4 peaks in global analysis and a significant Period-4 peak in the histograms, whereas H6 has no 1:4 peak in global analysis and a barely discernible Period-4 peak in the histogram.

### Effects of amiodarone

Three hearts (H3, H4, and H6) were on amiodarone, a multi-channel membrane active antiarrhythmic medication (*Table 1*). According to the histograms in *Figure 7*, amiodarone’s primary effect is promoting the 1:1 peak, i.e. increasing the size of the areas without alternans.

### Electrophysiological characteristics of higher-order areas

**Figure 8:**
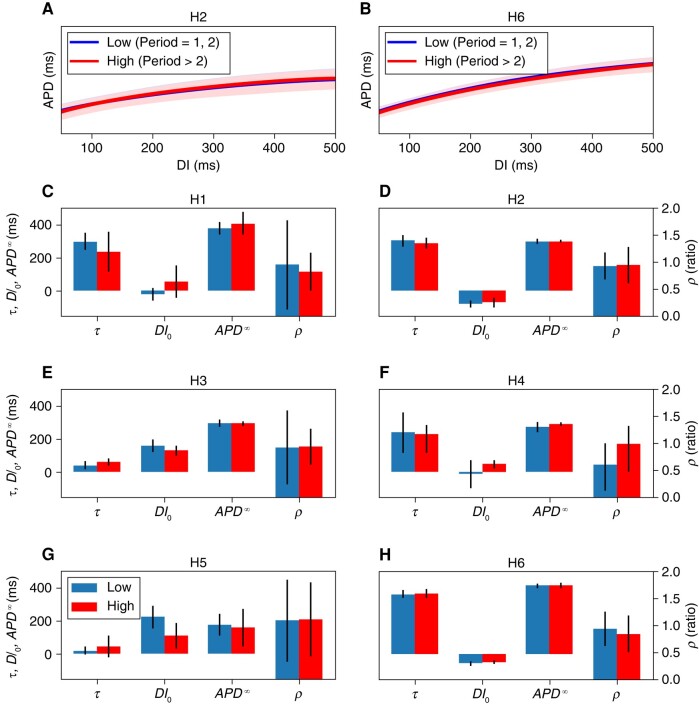
The electrophysiological characteristics of areas with different periodicities. The top row (*A* and *B*) shows the overlapped restitution curves for two hearts. The curves depict regions of Periods 1 and 2 (low) and areas of Periods 3–8 (high). The restitution curves are so similar that it is difficult to separate the two curves. (*C*–*H*) The electrophysiological properties (restitution-curve parameters and conduction velocity) between each heart’s low and high periods regions. Restitution curves are parameterized by $\mathrm{AP}D^{\infty }$, $DI_{0}$, and *τ* (see the Methods section). The ratio of conduction velocity at fast pacing rates (the last three recordings before fibrillation) to the conduction velocity during slow pacing (remaining recordings) is denoted *ρ*.

*Figure 8* compares the electrophysiological properties between low and high periodicity areas (low periodicity is defined as Periods 1 and 2, and high periodicity as Periods 3 and higher). There is no significant difference in the baseline electrophysiological characteristics between the low and high periodicity regions, which are essentially indistinguishable. In other words, *the underlying electrophysiological properties, measured by the restitution curve and local conduction velocity, do not predict the dynamics when pacing at faster rates*.

## Discussion

We report what we believe to be the first detection of stable Period-4, and intermittent Periods 6 and 8 in human cardiac tissue during fast stimulation. There is a large degree of heterogeneity in the distribution of higher-order periodic areas among the hearts and in different regions of the same heart. However, periodicities larger than 2 (i.e. beyond classic alternans) were detected in all six hearts, and they are likely a common occurrence in severely diseased human hearts at sufficiently fast pacing rates. Moreover, we observed the co-existence of higher-period regions with lower-period regions (Period-1 and Period-2), suggesting the existence of an interval when portions of the ventricles are already electrically unstable without florid VT or VF.

We observed no significant baseline electrophysiological differences between areas with Period-2 and areas with Periods 4, 6, and 8. Specifically, restitution curves remain the same. Therefore, the baseline restitution properties do not predict the ultimate pre-VF dynamics.

These results have mechanistic implications. Two known mechanisms for generating alternans and higher-order periodicities are voltage-driven (dependent on the restitution properties) and calcium-driven.^7,24^ Based on the observed independence of the local dominant period from the restitution properties, we infer that the oscillation of the calcium machinery and excitation-contraction coupling is the primary driver of higher-order dynamics in human hearts. This result is consistent with both theoretical works, showing that monotonically increasing restitution curves (as in human hearts) cannot generate periods >2,^25^ and electrophysiological studies during cardiac surgery, reporting that restitution properties cannot distinguish between alternans-susceptible and alternans-resistant regions.^26^

As mentioned, one of the main motivations behind this study was to develop a theoretical framework for possible substrate-directed ablation of VF. Our results, as relevant to VF ablation, are mixed. On the one hand, detecting higher-order periodicities in human hearts, the spatial heterogeneity of these regions, and the co-existence of chaotic pockets and normal rhythm are promising evidence that target regions exist. On the other hand, we noted that the restitution properties and conduction velocity are not predictors of these areas; therefore, it is unclear how we can locate these targets during ablation. Our results do not preclude the possibility that local differences in calcium cycling properties can predict the site of higher-order dynamics; however, no clinically feasible technique exists to measure these characteristics.

We did observe larger non-alternating areas (Period-1) in amiodarone-treated hearts relative to higher-order periodicities, which points to the membrane-stabilizing effects of amiodarone. Nevertheless, this effect is partial, and the amiodarone-treated hearts still exhibit higher-order periodicities.

Transplant recipient hearts used in this study are very diseased and electrophysiologically different from normal human hearts. Previous experiments have shown spatial variability in the electrophysiological characteristics in severely diseased hearts. For example, the ischaemic scar border zone has prolonged APD.^27^ Similarly, diffuse fibrosis and gap-junctional dysfunction in non-ischaemic cardiomyopathy results in conduction velocity slowing and heterogenous prolongation of APD, both of which are arrhythmogenic.^28,29^ The effects of the ventricular substrate abnormalities on the genesis of higher-order dynamics are still unclear and require further study; however, heterogenous scarring in heart failure may be responsible for the observed variability of the higher-period regions.

Detecting higher-order periodicities in human hearts may have practical implications beyond its relevance to ablation. If a practical method to detect Period-4 from clinical recordings (e.g. surface electrocardiogram) is developed, it might fix the main shortcoming of T wave/APD alternans in the form of low positive predictive value for malignant ventricular arrhythmias.^30^

### Limitations

Human recipient hearts used in this study are very heterogeneous with variable age, sex, underlying disease, pre-transplant ejection fraction, exposure to different antiarrhythmic medications, and the use of mechanical circulatory support before transplantation. In addition, the logistical problems in performing the experiments prevent using a large sample size. The small sample size and heterogeneous hearts make it difficult to perform accurate statistical analysis. While the study’s primary finding, i.e. the detection of higher-order periodicities in human hearts, is robust, the secondary results, primarily related to the mechanistic differences, are less confident and should be considered mainly as hypothesis generation rather than confirmatory. In addition, we cannot prospectively control for confounding factors, like exposure to amiodarone and inotropes.

The results from diseased transplant recipient hearts may not apply to normal hearts. Nevertheless, these are precisely the hearts prone to malignant arrhythmias that would benefit from ablation. The results obtained from recipients’ hearts are likely more clinically relevant than studies done on normal hearts.

Detecting higher-order periodicities is challenging and requires advanced signal processing and specialized algorithms (see the Supplementary material online). Moreover, the spatiotemporal distribution of the higher-order dynamics is complex, and optical mapping is not a viable clinical method for the foreseeable future. Therefore, translating the results of this study to clinical practice can be difficult. Nevertheless, a subset of the techniques discussed here may be practical. Specifically, high-density mapping of Period-4 regions using multipole catheters is within the reach of modern clinical electrophysiology mapping systems.

## Conclusions

Focal areas of higher-order periods can occur in diseased human hearts under fast pacing. The underlying electrophysiological characteristics, as measured by the restitution properties and conduction velocity, are not predictive of the localization of these areas.

## Supplementary Material

euad350_Supplementary_Data

## Data Availability

Certain processed and anonymized data products and the signal processing code (in Julia programming language) will be shared on reasonable request to the corresponding author.
