# Supplementary material for: Complex repolarization dynamics in ex vivo human ventricles are independent of the restitution properties
Source: Europace. 2023 Nov 25;25(12):euad350. doi: 10.1093/europace/euad350 (PMC10751849; doi:10.1093/europace/euad350)
Supplement: euad350_Supplementary_Data [file euad350_supplementary_data.pdf]

Supplement A: Spatial Filtering

We use a variational method to perform spatial filtering to better control and improve the definition of wavefronts. Let $g(x, y)$ be the normalized fluorescent value (in the 0 to 1 range with 1 corresponding to peak depolarization) of the pixel at the position x and y, and $f(x, y)$ be the desired smoothed value. We define two discrete energy functions. First, the smoothness function,

$$S = \sum_{i,j} (f(x + 1, y) - f(x, y))^2 + (f(x, y + 1) - f(x, y))^2.$$

Next, the data energy function is defined as

$$D = \sum_{i,j} (f(x, y) - g(x, y))^2.$$

Our goal is to minimize the total energy, $E = S + \lambda D$, where λ is a super-parameter. Because of the way energy functions are defined, the total energy is in quadratic form. The resulting minimization problem reduces to solving a sparse linear system, which can be done efficiently using the gradient conjugate method.

Supplement B: Global Analysis

The key to global analysis is to remove the effects of wave propagation to distill the dynamics to a few aggregate channels focused on the repolarization phase. We shift the signals to align the upstrokes (Figure 1, panel A is unshifted signals, and B is frame shifted). Because our signals were recorded while pacing the heart at a stable rate, frameshifting is possible.

The core of our global processing routines is dimensionality reduction. We use the standard Principal Component Analysis (PCA) method. Each frame-shifted signal cube is flattened into a two-dimensional matrix (one temporal and one spatial dimension) and subjected to the truncated Singular Value Decomposition.

The top few (~5-10) principal components capture most of the dynamics (Figures 1, panels C and E show the top two principal components, marked as W1 and W2). Finally, we generate the spectrograms of the top principal components (Figures 1, panels D and F). The frequency is normalized to the frequency of the driving stimulation; therefore, the 1:1 peak corresponds to the principal action potential propagation. We are mainly interested in the sub-harmonics of the 1:1 peak. The 1:2 peak (located at exactly half the driving frequency) is a sign of period-2 alternans. Similarly, the 1:4 peak is a marker of the period-4 oscillation in the repolarization phase.

Supplement C: Local Analysis

We discuss the combinatorial algorithm to find the optimal periodicity of each beat in a given input sequence. The algorithm is applied to each valid pixel in the input data and outputs a period map, where the dominant periodicity of each pixel is marked.

For each pixel, the input to the algorithm is a sequence of n beats separated by the upstroke times. Let $d(i, j,)$ be a *distance* function that returns a non-negative real value, quantifying the difference between beats i and j. We assume that $d(i, j)$ satisfies the axioms of a distance (or metric) function, meaning that $d(i, i) = 0$, $d(i, j) = d(j, i)$, and $d(i, j) + d(j, k) \leq d(i, k)$. In this paper, we define $d(i, j)$ to be the mean squared difference between beats i and j. In other applications, $d(i, j)$ may be defined as $|APD(i) - APD(j)|$.

We start the discussion by presenting the combinatorial algorithm to detect period-2 alternans. Our task is to classify each beat in the input sequence as one of two classes A and B. For example, A can be the long APD beats and B the short APD ones. A stable alternating sequence can be written as $ABABAB \cdots$. For such a sequence, we can simply assign A to the odd beats and B to the even beats. However, the input sequence may glitch (e.g., two adjacent beats are both short APD) such that the odd/even algorithm fails to work. This problem is especially relevant to higher-order periodicity, where glitches and frameshifts are the rules rather than the exception. The combinatorial algorithm is designed to overcome this shortcoming of simple periodicity detection algorithms.

We find the optimal assignment by setting a weighted directed graph in such a way that the shortest path between the starting vertex (beat 1) and the last vertex (beat n) reveals the optimal assignment (Figure S1, panel A). Let $G = (V, E)$ be a graph composed of n vertices V (corresponding to n beats) and $m > n$ edges E. For the detection of period-2, we set up two edge types. An edge of the first type connects

vertex i to vertex $i + 1$ with weight $d(i, i + 1)$. An edge of the second type connects i to $i + 2$ with weight $d(i, i + 2) + d(i + 1, i + 3) + \eta w$, where w is the mean distance between two adjacent vertices, i.e., $w = \overline{d(i, i + 1)}$, and η is a super-parameter. The second term is for regularization to prevent spurious high-order detection by favoring shorter periods. The optimal solution is found by assigning A to each vertex on the shortest path from 1 to n and B to the vertices not on the shortest path.

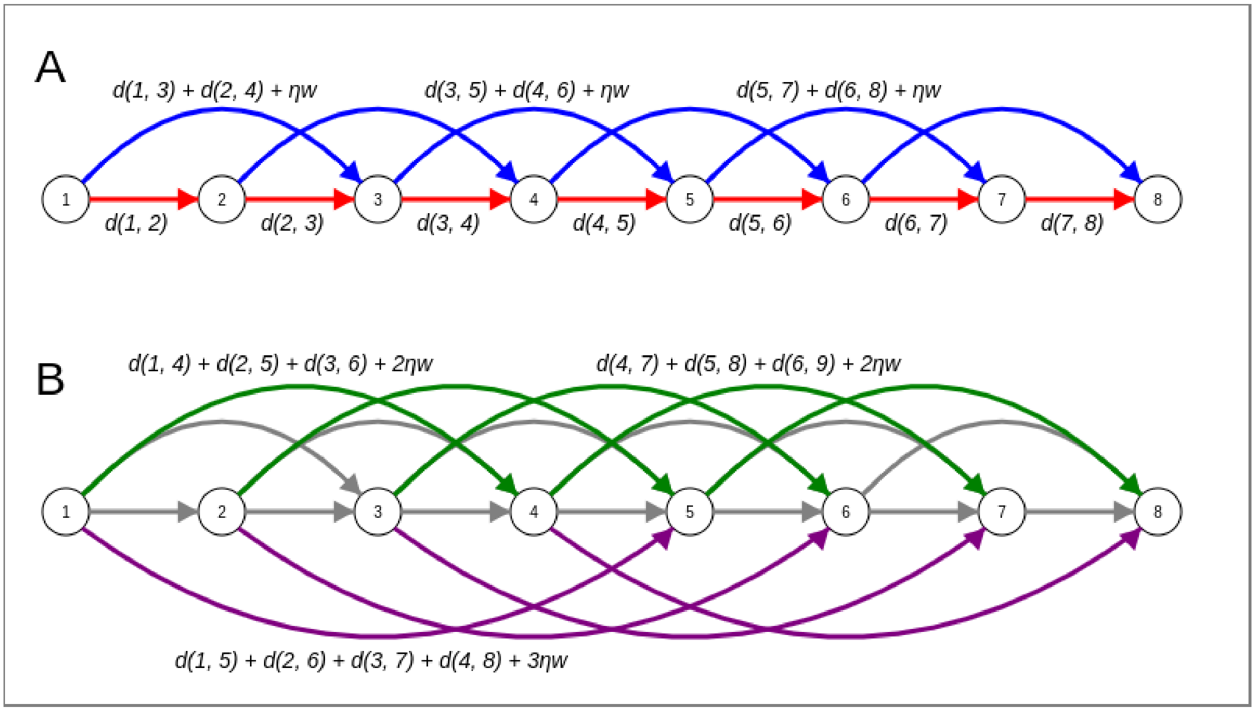

S1. Schematics of the combinatorial algorithm for local analysis. The graph used to detect alternans, showing $i \to i + 1$ (red) and $i \to i + 2$ (blue) edges (**A**). An extended graph to detect up to period-4 oscillations. In addition of the links in A, it also has $i \to i + 3$ (green) and $i \to i + 4$ (purple) edges (**B**).

For detection of period-4, we expand the possible classes of assignment to $\{A, B, C, D\}$. Now, an ideal input sequence is $ABCDABCDA \cdots$. We can modify the algorithm above by adding edges of type $i \to i + 3$ and $i \to i + 4$ to detect periods up to 4 (Figure S1, panel B).

Finally, we extend the algorithm to detect all periodicities up to a maximum p. In this paper, we set $p = 8$. We add p outgoing edges to each vertex i as follows. For $1 \leq l \leq p$, we add an edge $i \to i + l$ with weight $\sum_{j=0}^{l-1} d(i + j, i + j + l) + \eta(l - 1)w$. We find the shortest path from 1 to n, say $1 \to v_1 \to v_2 \to \cdots \to v_k \to n$. Then, the sequence $D = (v_1 - 1, v_2 - v_1, \cdots, n - v_k)$ encodes the periodicity of the signal. The predominant periodicity of each pixel is defined as the mode of D.
